# The Impact of Clostridioides Difficile Infection in Hospitalized Patients: What Changed during the Pandemic?

**DOI:** 10.3390/diagnostics12123196

**Published:** 2022-12-16

**Authors:** Alina Boeriu, Adina Roman, Daniela Dobru, Mircea Stoian, Septimiu Voidăzan, Crina Fofiu

**Affiliations:** 1 Gastroenterology Department, University of Medicine Pharmacy, Sciences, and Technology “George Emil Palade” Targu Mures, 540142 Targu Mures, Romania; 2 Gastroenterology Department, Mureș County Clinical Hospital, 540103 Targu Mures, Romania; 3 Intensive Care Unit Department, University of Medicine Pharmacy, Sciences, and Technology “George Emil Palade” Targu Mures, 540142 Targu Mures, Romania; 4 Intensive Care Unit Department, Mureș County Clinical Hospital, 540103 Targu Mures, Romania; 5 Epidemiology Department, University of Medicine Pharmacy, Sciences, and Technology “George Emil Palade” Targu Mures, 540142 Targu Mures, Romania; 6 Internal Medicine Department, Bistrița County Hospital, 420094 Bistrița, Romania

**Keywords:** *Clostridioides difficile*, SARS-CoV-2, outcomes

## Abstract

(1) Background: *Clostridioides difficile* (*C. difficile*) and SARS-CoV-2 coronavirus represent significant health threats. Our study focused on the impact of concurrent infections on patient outcomes against the backdrop of changes imposed by the pandemic. (2) Materials and methods. We performed a retrospective analysis and included patients diagnosed with CDI who were admitted in our hospital before and during the pandemic. We compared patient exposure to risk factors for CDI in both groups and patient negative outcomes: need for ICU care, prolonged hospitalization, organ failure, toxic megacolon, and death. (3) Results. Overall, 188 patients were included, of which 100 had CDI (the pre-pandemic group), and 88 patients presented both CDI and COVID-19 (the pandemic group). Patients in the pandemic group were significantly older, with a higher Charlson Comorbidity Index (CCI) and a greater exposure to antibiotics and corticosteroids, and were more likely to develop organ dysfunction, to require ICU care and have prolonged hospitalization. The severity of COVID-19, leukocytosis and increased D-dimer levels were indicators of poor prognosis in the pandemic group. Higher CCI scores and leukocytosis increased the risk for negative outcomes in CDI alone patients. (4) Conclusions. The study highlights the negative impact of associated infections on patient outcome. The severity of COVID-19 directly influences the prognosis of patients with concurrent infections

## 1. Introduction

The emergence of novel coronavirus (severe acute respiratory syndrome coronavirus 2 SARS-CoV-2) and the spread of the infection at the global level posed new challenges and health care concerns. Both *Clostridioides difficile* (*C. difficile*) and SARS-CoV-2 virus are major public health threats with potentially severe consequences, especially among elderly patients with chronic disorders.

Coronavirus disease 2019 (COVID-19) presented as a complex, systemic disease, comprising a wide spectrum of clinical manifestations involving the respiratory tract, among others: gastrointestinal, cardiovascular, neurological, renal, dermatological, and coagulation disorders have all been described among SARS-CoV-2 infected patients [1,2,3,4,5,6,7].

*C. difficile* infection (CDI) showed a growing tendency in years before the pandemic, resulting in an increased length of hospitalization and health care-associated costs, in addition to a higher mortality rate [8,9]. Older age, antibiotic exposure and hospitalization have been described as major risk factors for *C. difficile* acquisition [10,11,12]. Other risk factors for CDI are immunosuppression, prolonged PPI use, chronic kidney disease, use of nasogastric tubes, and gastrointestinal surgery [13,14,15,16,17,18,19,20]. The new onset of unexplained diarrhea, especially in vulnerable categories of patients, with more than three unformed stools in 24 h, requires CDI testing for appropriate therapy [21].

During the pandemic, gastrointestinal symptoms were related to SARS-CoV-2 virus, which binds to angiotensin converting enzyme 2 receptors (ACE 2) through spike protein, enters into epithelial cells, activates immune responses and promotes tissue injury. Since ACE 2 receptors are present not only in the respiratory tract but also in the cardiovascular system, gastrointestinal tract, urogenital system, liver and gallbladder, and in the nervous system, COVID-19 may evolve with multi-organ damage [22]. Nausea, vomiting, diarrhea and abdominal pain have all been reported in COVID-19 patients [23,24,25]. Digestive complaints may occur initially in the absence of respiratory symptoms in some cases, leading to a diagnostic delay of SARS-CoV-2 infection [26,27].

In clinical practice symptoms that usually correspond to CDI may overlap with digestive complaints secondary to SARS-CoV-2 virus, therefore during the pandemic some authors recommended testing suspect cases for both infections [28]. According to other authors, the impaired immune system in severe COVID-19 and alteration of gut microbiota composition may be responsible for *C. difficile* acquisition [29,30]. As viral RNA was detected in stool samples, it is possible that SARS-CoV-2 can also be transmitted by the fecal-oral route [31]. This represents a concern when CDI developed in a patient with COVID-19, and potentially facilitated the spread of both pathogens by watery stools, forcing the intensification of infection control measures [29].

In recent studies, authors have assessed the incidence of gastrointestinal pathogens in COVID-19 patients presenting with recent onset diarrhea, and concluded that *C. difficile* infection was the significant contributor to diarrhea in COVID-19 patients, although initially, incidence of CDI was thought to be significantly lower compared to the pre-pandemic period. The main risk factor was antibiotic treatment, and the outcome of COVID-19 patients with *C. difficile* was significantly unfavorable, with an increased rate of admission to the intensive care-unit, or an increased need for mechanical ventilation [32,33]. A significant lower number of studies have focused on the comparison of *C. Difficle* positive patients before the pandemic period and during the pandemic period, with an associated SARS-CoV-2 infection, although their hospitalization period, treatment administered and risk factors were similar.

The primary aim of our study was to perform a comparative analysis of two distinct groups of patients diagnosed with CDI, who were hospitalized before and during the COVID-19 pandemic, in order to detect the impact of associated infections on patient outcomes and mortality. The secondary endpoints were to compare the exposure to traditional risk factors for *C. difficile* acquisition in both groups and to determine prognostic factors for severe progression of the disease.

## 2. Materials and Methods

We conducted a retrospective study in a high-volume tertiary university hospital and analyzed two distinct periods: pre-pandemic period (January 2019–February 2020) and pandemic period (March 2020–May 2021). Data were collected from medical records of patients hospitalized in several departments of our hospital: the gastroenterology department, department of infectious diseases, department of internal medicine, surgery department and intensive care unit (ICU). All the patients included in our study were diagnosed with CDI on admission or during hospitalization. Since March 2020 our hospital has been converted to treat exclusively COVID-19 patients, and the second group included patients with both SARS-CoV-2 and *C. difficile* infection.

Patients who met clinical criteria (new onset diarrhea, consisting of three or more unexplained loose stools in 24 h) and laboratory criteria (positive glutamate dehydrogenase GDH test and toxin enzyme immunoassay EIA (bioMerieux, Marcy-l’Étoille, France)) for CDI were included. During the COVID-19 period, all the patients had a positive reverse transcription-polymerase chain reaction (RT-PCR) test result for SARS-CoV-2 from a nasopharyngeal swab (CFX96 Real-Time PCR Systems (Bio-Rad, Hercules, CA, USA)). The study was approved by the Ethical Committee of the hospital.

We analyzed demographic data, clinical data, comorbidities and Charlson Comorbidity Index (CCI), laboratory parameters (complete blood count, C reactive protein CRP, ferritin, D-dimer, creatinine, albumin, liver tests), CDI data (first episode or recurrence), and risk factors for CDI: exposure to high-risk medication within three months prior to current admission and during hospital stay (antibiotics, PPI or histamine-2 blockers treatment, steroids use), recent hospitalization for medical illness and recent surgery (in the 12 weeks prior to CDI diagnosis) and immunosuppression (malignancy, autoimmune diseases, immunosuppressive therapy). The severity of COVID-19 was categorized as mild (symptoms without pulmonary lesions on imaging), moderate disease (pulmonary lesions on imaging, oxygen saturation ≥ 94% on ambient air), severe (respiratory rate ≥ 30/min, oxygen saturation ≤ 93% on ambient air, or lung infiltrates > 50%), and critical disease (respiratory failure, requirement of mechanical ventilation, shock).

We compared the evolution of the disease and assessed predictors for severe progression in two groups of patients: CDI-alone patients before the pandemic and CDI-COVID-19 patients during the pandemic. In the second group, we analyzed patients with mild/moderate COVID-19 versus patients with severe/critical COVID-19. We considered the following negative outcomes: longer hospital stays, the progression to organ failure, the development of toxic megacolon, the requirement for ICU care and death.

### Statistical Analysis

The data collected were coded, processed, and analyzed with SPSS version 22 (Statistical Package for Social Sciences) (IBM, SPSS Inc., Chicago, IL, USA). Comparisons between the two groups with categorical variables were performed by Chi-Square test (or Fisher‘s exact test). To compare the two groups with gaussian distributed quantitative variables, an independent samples (student’s) *t*-test was used, while for non-gaussian distributed data, the Mann–Whitney U-test was used. Univariate and multivariate logistic regression analyses were used to identify the dependent and independent risk factors predictors for severe outcome. A two-sided error level of *p* < 0.05 was considered statistically significant.

## 3. Results

### 3.1. General Analysis of Patient Characteristics

We enrolled a total number of 188 patients with CDI, of whom 100 were hospitalized during the pre-pandemic period (CDI alone patients) and 88 patients during the COVID-19 pandemic (CDI-COVID-19 patients). Patient demographic, comorbidities, risk factors for CDI, laboratory characteristics and negative outcomes are summarized in Table 1. CDI-COVID-19 patients were older than patients in the non-COVID group (*p* = 0.025), while gender showed no significant difference between the two groups. Charlson comorbidity index (CCI) was assessed for each patient and it was obvious that almost all individuals in CDI-COVID-19 group had multiple associated diseases and higher CCI scores were mainly detected in COVID-19 group: 83% versus 59% in CDI alone patients (*p* = 0.0031). Hospitalization in the previous 30 days before the current admission for surgical or medical conditions was recorded in 64% of non-COVID patients, compared to 39.8% in COVID group (*p* = 0.001). There was no significant difference between the two groups regarding recent surgical history (*p* = 0.064).

### 3.2. High-Risk Medication for CDI Analysis

We compared the exposure to high-risk medication for CDI in both groups. From 100 patients in the pre-pandemic group, 66% were exposed to antibiotics (44% of them had a positive culture that confirmed an infection), 21% to PPIs, 50% to H2 blockers, and 13% to steroids. A significantly greater exposer to antibiotics was noticed in the COVID-19 group: 76 patients (86.3% vs. 66%, *p* = 0.0021) received antibiotics, most of them (77.63%) in the absence of a bacteriological confirmation of infection, while the rest had positive respiratory culture (18.42%), positive urine culture (17.1%) and/or positive blood culture (2.63%) (Table 2). Significant higher steroid use was detected in the CDI-COVID-19 group (58 patients, 65.9% vs. 13%, *p* = 0.00001), whilst 16 patients (18.2%) received PPI.

### 3.3. Paraclinical Assessment Analysis

When analyzing laboratory parameters, hemoglobin showed a significant drop in CDI-alone patients (*p* = 0.02), whilst leucocytes and platelet count showed no significant difference between the two groups. However, significant differences were observed in inflammatory marker values: CRP showed a notable raise in the pre-pandemic group (*p* = 0.027), while ferritin levels were higher in the COVID-19 group (*p* = 0.0001). There was no significant difference between the two groups in regards to albumin and creatinine levels (Table 1).

### 3.4. Hospitalization Period and Evolution of CDI Patients Analysis

The median length of hospital stay in CDI-COVID-19 patients was 15 days, significantly longer compared to CDI alone patients (8 days, *p* = 0.0001). Associated *C. difficile* and COVID-19 infections were correlated with a significantly higher rate of ICU admission (33% vs. 13%, *p* = 0.001), organ failure (77.2% vs. 24%, *p* = 0.0001), and mortality (23.9% vs. 13%), when compared to the CDI alone group. One patient developed toxic megacolon and required surgical intervention in the pre-pandemic group, and no cases of toxic megacolon were found in CDI-COVID-19 group.

### 3.5. Associated Risk Factors Analysis

We performed a univariate analysis to identify risk factors for a fatal outcome in both groups (Table 3). Associated comorbidities such as liver cirrhosis and diabetes mellitus were identified as risk factors for fatality in the pre-pandemic group. Proton pump inhibitors use (58.6%, *p* = 0.01) and steroid treatment (71.4%, *p* = 0.0012) were correlated with mortality in CDI-COVID-19 patients, while treatment with H2 blockers (92.3%, *p* = 0.032) was significantly associated with death in CDI-alone patients. Severe progression to organ dysfunction has been linked with increased mortality in both groups. ICU admission, sepsis, cardiac failure, liver failure, and kidney failure were risk factors for death in the pre-pandemic group, while respiratory insufficiency and multiple organ failure (MOF) were significantly associated with mortality in the pandemic group (*p* < 0.05). Recurrent CDI was recorded in 17% of CDI alone patients, compared to 4.5% in COVID-19 group (*p* = 0.007). However, recurrent CDI did not represent a risk factor for death.

### 3.6. Risk Factors for Negative Outcome Analysis

We performed a multivariate logistic regression analysis to estimate the risk for negative outcomes in CDI patients with COVID-19 (Table 4). Variables of interest for multivariate analysis included age, gender, comorbidities, CCI, COVID-19 severity, and laboratory parameters. The severity of COVID-19 (OR 3.42; 95% CI: 1.41–8.31, *p* = 0.0064) and leukocytosis (OR 16.01; 95% CI: 1.76–145.48, *p* = 0.0137) increased the risk for prolonged length of hospital stay. D-dimer > 0.5 μg/mL on admission (OR 6.26; 95% CI: 1.41–27.81, *p* = 0.0157) significantly correlated with the need for ICU care, while severe/critical COVID-19 significantly increased the risk for organ failure (OR 8.59; 95% CI: 2.54–28.98, *p* = 0.0005) and death (OR 25.19; 95% CI: 3.18–199.5, *p* = 0.002). Other factors such as advanced age (>65 years old), higher CCI score, anemia, and hypoalbuminemia were not predictors for severe outcomes in the multivariate analysis in the CDI-COVID-19 group.

### 3.7. Associated Severity of COVID-19 Analysis

Among CDI-COVID-19 patients, 50 patients (56.8%) presented severe or critical COVID-19 illness. We found that patients with severe/critical COVID-19 were at the highest risk for prolonged hospitalization (OR 3.79; 95% CI, 1.32–10.84, *p* = 0.01), organ dysfunction (OR 4.97; 95% CI, 1.36–18.12, *p* = 0.01), and death (OR 16.40; 95% CI, 1.97–136.38, *p* = 0.009) (Table 5).

By analyzing risk factors for poor outcomes in the CDI-alone group, we found that a higher comorbidities score (CCI > 5) increased the risk for ICU admission (OR 5.27; 95% CI, 1.31–17.33, *p* = 0.002), organ failure (OR 11.88; 95% CI, 1.75–80.66, *p* = 0.01), and death (OR 10.93; 95% CI, 1.16–102.98, *p* = 0.03). CDI patients with leukocytosis >15.000 presented a higher risk for negative outcomes: ICU admission (OR 5.16; 95% CI, 2.40–9.81, *p* = 0.003), organ failure (OR 3.97; 95% CI, 1.27–12.41, *p* = 0.01), and death (OR 7.19; 95% CI, 1.49–34.67, *p* = 0.01). CCI ≥ 5 and anemia (Hgb < 9 g/dL) were negatively associated with prolonged hospitalization (OR 0.30; 95% CI, 0.08–1.13, *p* = 0.05 and OR 0.17; 95% CI, 0.06–0.46, *p* = 0.0004 respectively), since many patients with multiple comorbidities likely died within the first days of hospitalization. (Table 6).

## 4. Discussion

Management of various diseases during the pandemic had to be adapted to the new conditions imposed by the rapid spread of the SARS-CoV-2 virus. Data collected from COVID-19 patients provided valuable information for clinical practice. Our study demonstrates that CDI in COVID-19 patients was associated with a poor prognosis. Patients with CDI and COVID-19 were older, had associated comorbidities, and were more frequently exposed to antibiotic and steroid treatment.

Many previously published studies have compared CDI incidence before and during the COVID-19 pandemic and some contradictory results have been obtained. Antibiotic stewardship, rigorous hand hygiene, environmental cleaning and disinfection, and the isolation of CDI cases in separate wards were some of the measures adopted to limit the risk of infections within the hospital in the pre-pandemic period. All these measures were intensified during the pandemic, with a positive impact on healthcare-associated infections, including CDI [34,35].

Although in our study we did not calculate the incidence of CDI, we noticed that in a similar time frame (14 months), 100 patients were diagnosed with CDI before the pandemic, and 88 patients during the COVID-19 pandemic. The danger of CDI for hospitalized patients is far from negligible, even with the restrictions imposed by the pandemic. Previously, Bentivegna et al. reported a significantly lower incidence of healthcare-associated CDI (HA-CDI) in 2020 in COVID-19-free medical departments, compared to previous years (2017, 2018, and 2019, respectively), although the incidence of HA-CDI was higher in COVID-19 departments, compared to COVID-19-free departments in 2020 [36]. Other authors reported a reduction in hospital-acquired CDI cases in those hospitals designated to treat COVID-19 patients [37]. Yadlapati et al. noticed slightly higher CDI rates in COVID periods compared to the non-COVID period, but without statistical significance. Significantly higher CDI rates were detected during COVID peak periods, potentially due to increased antibiotic prescription [38]. Other authors pointed to the impact of CDI during the pandemic. Thus, Lewandowski et al. found a significantly higher CDI incidence, compared to the pre-pandemic period (10.9% versus 2.6%, *p* < 0.001), whilst Granata et al. showed, in a systematic review and meta-analysis, that CDI incidence rates in COVID-19 patients ranged from 1.4 to 4.4 CDI cases per 10,000 patient-days [39,40].

Although the implementation of isolation measures, restrictions for visitors, limited hospital consultations and surgical procedures could explain the reduced CDI incidence reported by some authors, the increased antimicrobial prescription and prolonged hospitalization in severe COVID-19 could have had the opposite effect. Additionally, it is possible that an underestimation of the real number of CDI cases during the pandemic resulted from a lower rate of *C. difficile* testing in patients with digestive complaints, most of them being attributed to COVID-19 infection [40].

Some drugs used in the treatment of COVID-19 have a known negative effect on gastrointestinal tract. Antibiotic usage was increased during the pandemic, even in the absence of a microbiological confirmation of bacterial infection [41,42]. In a review of nineteen clinical studies, including 2834 hospitalized patients with COVID-19, the mean rate of antibiotic use was 74%, with fluoroquinolones, macrolides and cephalosporins being the most commonly prescribed, even in non-severe and non-critical cases [43]. In general, antibiotic prescriptions for all COVID-19 patients during the pandemic, mostly indicated to control bacterial co-infections and superinfections, were estimated to exceed 70% of all COVID-19 patients [44,45].

The results of our analysis reflect this tendency. A significant percent of CDI-COVID-19 patients received antibiotics (86.3%, *p* = 0.0021), with a bacteriological confirmation of infection in only 22.37% of them. We detected an increased in cephalosporins (57.9%), carbapenems (12.5%), penicillin (6.8%), and quinolones (4.5%) use among CDI patients during the COVID-19 pandemic. In the pre-pandemic period, 66% of CDI patients were exposed to antibiotics, most of them (67%) with a positive culture confirming infection.

A consequence of increased antibiotics prescription in COVID-19 is gut dysbiosis, that creates a favorable environment for *C. difficile*. However, Hawes et al. reported a stable incidence of CDI during the first peak of the pandemic, despite increasing clindamycin, cefotaxime, ceftriaxone, ceftazidime, and quinolone consumption [46]. Similarly, other authors reported a decreased incidence of CDI that proved the positive effect of intensified cleaning measures together with reduced patient mobility [47].

Severe cases of CDI following antibiotic use for COVID-19 pneumonia, which progressed to toxic megacolon and death, were reported in the literature [48,49]. None of the 88 CDI-COVID-19 patients included in our study developed toxic megacolon. The unique case of toxic megacolon who required colectomy was reported in the pre-pandemic group. Our analysis proved that the immediate unintended consequence of antibiotic exposure in COVID-19 patients may be the acquisition of CDI. In the long term, the potential risk of antimicrobial resistance caused by antibiotics and disinfectants overuse during the pandemic, including the possible selection of resistant *C. difficile* strains in hospitals and in the community, is a matter of great concern [50].

Recent hospitalization in CDI patients was mainly detected in the pre-pandemic group (64% vs. 39.8%, *p* = 0.001), which reflects the reduced hospitalization rate imposed by pandemic restrictions, with potentially beneficial consequences on *C. difficile* rate. Other authors showed that previous hospitalization and antibiotic use represent significant risk factors for CDI in COVID-19 [51]. Previous hospitalization, previous steroid use, and antibiotics during the hospital stay were identified as risk factors for CDI in COVID-19 by Granata et al., similar to our data. This underlines the importance of antimicrobial stewardship programs during the pandemic [52]. Steroids use along with antibiotics use was significantly higher among COVID-19 patients in our analysis: 58 patients (65.9%) received corticosteroids (*p* = 0.00001), and dexamethasone was prescribed in 53.4% of COVID-19 patients. In the pre-pandemic group, CDI patients were mainly exposed to acid-suppression medications (PPIs and histamine-2 receptor antagonists).

Univariate analysis showed that steroid therapy and PPI use were significantly correlated with death in CDI-COVID-19 group. The increasing risk for severe clinical outcomes (including admission to ICU, acute respiratory distress syndrome ARDS or even death) in COVID-19 patients exposed to PPI was previously reported by some authors, although other authors showed no significant risk for severe COVID-19 infection, need for hospitalization, or 30-day mortality in PPI users [53,54,55,56].

Corticosteroids have been included in therapeutic protocols of COVID-19, and dexamethasone showed positive results in COVID-19 patients who received oxygen supplementation or invasive mechanical ventilation [57]. As mainly severe cases of COVID-19 received steroids, this explains the significant positive correlation between steroid treatment and a fatal outcome, which our analysis reveals. Although some studies showed that corticosteroids increased the risk for CDI, contrary results were reported by other authors [58,59,60,61]. There was initially significant concern regarding CDI risk after increased glucocorticoids use in the COVID-19 pandemic, but Carlson et al. demonstrated in a recent report that corticosteroids significantly reduced the odds of developing CDI in patients who received antibiotics [62,63].

Regarding the outcomes of patients with associated *C. difficile* and SARS-CoV-2 infections, our data are consistent with previously published reports. Clinical trials proved that risk factors for increased morbidity and mortality in SARS-CoV-2 are advanced age, associated comorbidities (obesity, diabetes, hypertension, coronary artery disease), and the severity of COVID-19 (acute respiratory distress syndrome, septic shock) [64,65,66,67,68]. In our study, the older age group was CDI-COVID-19, and the median age was over 60 years in both groups. Higher CCI scores were mainly detected among CDI-COVID-19 patients (*p* = 0.00031), although in the multivariate analysis, CCI > 5 significantly increased the risk for negative outcomes (organ failure, need for ICU care, and death) in the CDI-alone group. Most deaths were registered in CDI-COVID-19 patients with associated comorbidities: cardiovascular diseases (90.5%), pulmonary disease (57.1%), obesity (43.1%), diabetes mellitus (33.3%), and kidney disease (28.6%). The severity of COVID-19 represents a risk factor for the development of organ failure, prolonged hospitalization, and death. The mortality rate was higher among CDI-COVID-19 patients compared to CDI alone patients (23.9% vs. 13%, *p* = 0.053), although without a statistical significance. Comparing the clinical evolution of hospitalized patients with CDI in the two periods, we noticed the obvious negative impact of associated infections. Patients with CDI and COVID-19 were more likely to develop organ dysfunction (77.2% vs. 24%, *p* = 0.0001), to require ICU care (33% vs. 13%, *p* = 0.001) and to have a longer length of hospitalization (15 days vs. 8 days, *p* = 0.001) compared with CDI alone patients, which implies higher healthcare costs.

Most studies have focused on the impact of CDI on the evolution of COVID-19 patients. The worse outcomes and longer hospital stays in patients with COVID-19 and CDI, as well as the recurrence of CDI after hospital discharge and subsequently CDI-related death, were reported by Granata et al., while Allegreti et al. showed a significantly higher mortality rate in patients with associated infections (80% in COVID-19 with CDI versus 12.2% in COVID-19 alone patients, *p* < 0.0001) [52,69]. Four of nine COVID-19 patients with concurrent CDI died during hospitalization according to a report by Sandhu et al. [28].

We included in the logistical regression analysis risk factors for negative outcomes in CDI. Older age, comorbidities, leukocytosis, renal failure, and hypoalbuminemia were previously reported in the literature as predictors for complicated CDI and increased mortality [70]. We showed that leukocytosis > 15,000 was a marker for prolonged length of hospital stay in CDI-COVID-19. D-dimer greater than 0.5 μg/mL on admission was significantly correlated with the need for ICU care. CDI-alone patients with leukocytosis presented a higher risk for poor outcomes (organ failure, ICU admission, and death).

Other studies previously demonstrated the importance of altered laboratory parameters in COVID-19. An increased leukocyte level and elevated C-reactive protein were identified as predictors of severe outcomes, and D-dimer greater than 0.5 μg/mL correlated with severe COVID-19 [71,72,73]. Older age, high Sequential Organ Failure Assessment (SOFA) score, and D-dimer greater than 1 μg/mL (OR 18.42; 95% CI: 2.64–128.55; *p* = 0.0033) on admission were identified as indicators for poor prognosis (death) in COVID-19 patients [67].

Our analysis brings attention to the importance of increased vigilance regarding hospital-acquired infections, in particular CDI. The vigilance can play a significant role in improving clinical outcome and justifies the implementation of intensive strategies for CDI control and management, particularly during the COVID-19 pandemic. Data were collected from different departments of the hospital to better reflect the impact of CDI in patients with multiple pathologies and exposed to various risk factors for *C. difficile* acquisition.

Our study has several limitations. Due to the retrospective nature of the analysis, data to calculate the incidence of CDI have not been recorded. The PCR test for the detection of *C. difficile* was not available in our hospital. In patients with suspected CDI and inconclusive GDH/toxins results, abdominal ultrasound or computed tomography specific aspect (thickness of bowel wall), or in some cases endoscopic and histopathological detection of pseudomembranes, supported the positive diagnosis. In addition, we did not follow up patients after discharge, and for this reason possible cases of *C. difficile* acquired during hospitalization and cases of recurrent CDI could have been omitted.

Although in the majority of the patients with COVID-19 we identified the exposure of patients to “classic” risk factors for CDI (such as antibiotics, PPIs, steroids, immunosuppression), a potential causal relationship between COVID-19 and *C. difficile* remains a subject of ongoing research [74]. Lakkasani et al. reported a case of a male patient with COVID-19 who developed CDI in the absence of exposure to antibiotics or PPI, and speculated on the potential deleterious effect on normal microbial flora and impaired immune response in COVID-19 disease which could facilitate CDI [75]. We detected 7 CDI cases with no antibiotic or PPI exposure in our COVID-19 group, with no immunosuppression, although we did not perform a comparative analysis with similar cases in the pre-pandemic. The small sample size did not allow us to draw reliable conclusions regarding the specific influence of COVID-19 on *C. difficile* acquisition.

## 5. Conclusions

In summary, our study underlines the particularities of CDI in hospitalized patients in light of the changes imposed by the COVID-19 outbreak: intensified rules and personal training for infection control, lower hospitalization rate, and increased antibiotic and steroid use. Associated CDI and SARS-CoV-2 infections increased the risk for patients, most of them of advanced age with multiple comorbidities, for severe disease progression, and increased the burden of health care costs resulting from higher ICU admission rates and longer hospital stays. Higher CCI scores and leukocytosis are predictors for poor clinical outcome in CDI. In the pandemic period, the severity of COVID-19 directly influenced the prognosis of patients with concurrent infections.

## Figures and Tables

**Table 1:** Patient characteristics in both groups.

Characteristic	CDI-COVID-19 Patients (*n* = 88)	CDI Patients (*n* = 100)	*p* Value
Age (years)			
mean ± SD	69.56 ± 12.389	64.84 ± 15.78	0.025 *
Sex, no (%)			
Female	42 (47.7%)	51 (51%)	
Male gender	46 (52.3%)	49 (49%)	0.654 **
Charlson comorbidity index, no (%)			
0	3 (3%)	10 (10%)	
1–2	1(1%)	7 (7%)	0.0031 **
3–4	11 (13%)	24 (24%)	
≥5	73 (83%)	59 (59%)	
Previous hospitalization, no (%)	35 (39.8%)	64 (64%)	0.001 **
Recent surgery, no (%)	16 (18.2%)	9 (9%)	0.064 **
Medication exposure, no (%)			
Proton pump inhibitors	16 (18.2%)	21 (21%)	0.62 **
H2 RA	0 (0%)	50 (50%)	-
Antibiotics	76 (86.3%)	66 (66%)	0.0021 **
Corticosteroids	58 (65.9%)	13 (13%)	0.00001 **
Laboratory findings:			
Hemoglobin (g/dL), mean ± SD	12.29 ± 3.09	11.34 ± 2.36	0.020 *
White blood cell count (/μL), mean ± SD	12,798.20 ± 6708.15	11,921.11 ± 6451.56	0.37 *
Platelet count (/μL), mean ± SD	233,741.76 ± 107,770.27	266,612.05 ± 156,617.24	0.098 *
CRP (mg/L), median (min–max)	17.10 (0.02–283.56)	33 (0.05–302)	0.027 ***
Ferritin (μg/L), median (min–max)	965.4 (96–4893)	47.5 (3–147)	0.0001 ***
Albumin (g/L), median (min–max)	28.28 ± 5.47	26.54 ± 8.09	0.34 *
Creatinine (mg/L), median (min–max)	0.82 (0.5–6)	0.80 (0.4–10)	0.27 ***
Length of hospital stay (days), median (min–max)	15 (4–50)	8 (1–33)	0.0001 ***
Negative outcomes, no (%)			
ICU care	29 (33%)	13 (13%)	0.001 **
Organ failure	68 (77.2%)	24 (24%)	0.0001 **
Death, no (%)	21 (23.9%)	13 (13%)	0.053 **
Toxic megacolon, no (%)	0	1 (1%)	-
Discharged, no (%)	67 (76.1%)	87 (87%)	0.059 **
Recurrent CDI, no (%)	4 (4.5%)	17 (17%)	0.007 **

ICU: intensive care unit; CD: *Clostridioides difficile*; * t-student test (data were analyzed by mean ± SD); ** chi square (data were analyzed by number/percent); *** Mann Whitney test (data were analyzed by median/minimum-maximum).

**Table 2:** High-risk medication in CDI-COVID-19 group.

No (%)	88 (100%)
Antibiotics	
Prior to admission	15 (17%)
Total (prior to admission and during hospital stay)	76 (86.3%)
No bacteriologic confirmation	59 (77.63%)
Respiratory culture	14 (18.42%)
Urine culture	13 (17.1%)
Blood culture	2 (2.63%)
Type of antibiotic	
Cephalosporins	51 (57.9%)
Penicillins	6 (6.8%)
Fluoroquinolones	4 (4.5%)
Macrolides	2 (2.27%)
Carbapenems	11 (12.5%)
Corticosteroids	
Prior to admission	11 (12.5%)
Total (prior to admission and during hospital stay)	58 (65.9%)

No: number.

**Table 3:** Univariate analysis of risk factors for a poor prognosis (death) in both groups.

Covariate	CDI + COVID-19 Patients (*n* = 88)	CDI Patients (*n* = 100)	Chi Square Test
Sex			
Female	8 (38.1%)	7 (53.8%)	0.311
Male	13 (61.9%)	6 (46.2%)	
Comorbidities			
Diabetes mellitus	7 (33.3%)	6 (46.2%)	0.024
Cardiovascular disease	19 (90.5%)	4 (30.8%)	0.359
Pulmonary disease	12 (57.1%)	1 (7.7%)	0.083
Kidney disease	6 (28.6%)	1 (7.7%)	0.878
Cancer	3 (14.3%)	-	-
Cirrhosis	1 (4.8%)	5 (38.5%)	0.027
Obesity	38 (43.1%)	-	-
Medication exposure			
Proton pump inhibitors	21 (58.6%)	3 (23.1%)	0.01
H2 RA	3 (14.3%)	12 (92.3%)	0.032
Antibiotics	18 (85.7%)	12 (92.3%)	0.974
Steroids	15 (71.4%)	2 (15.4%)	0.0126
Recurrent CDI	1 (4.8%)	2 (15.4%)	0.956
Complications			
Admission in ICU	16 (76.2%)	12 (92.3%)	0
Respiratory insufficiency	21 (100%)	9 (69.2%)	0.003
Cardiac failure	9 (42.9%)	13 (100%)	0.001
Kidney failure	9 (42.9%)	7 (53.8%)	0.002
Liver failure	0 (0%)	6 (46.2%)	-
Sepsis	3 (14.3%)	8 (61.5%)	0.014
MOF	6 (28.6%)	1(7.7%)	0.009

CDI: *Clostridioides difficile* infection; H2 RA: histamine 2 receptor antagonist; ICU: intensive care unit; MOF: multiple organ failure.

**Table 4:** Multivariate analysis of risk factors for poor prognosis in CDI-COVID-19 group.

		ICU Admission			Longer Hospital Stays	
Covariate	OR	95% CI	*p* Value	OR	95% CI	*p* Value
Age > 65 years	0.88	0.33–2.34	0.79	1.32	0.52–3.31	0.54
Male gender	1.81	0.73–4.51	0.19	1.08	0.46–2.50	0.85
Comorbidities	2.25	0.34–14.83	0.39	0.79	0.18–3.37	0.75
Severe/critical COVID	-	-	-	3.42	1.41–8.31	0.0064
CCI ≥ 5	0.86	0.18–4.10	0.85	1.75	0.32–9.43	0.51
Hgb < 9 g/dL	2.97	0.38–22.87	0.29	0.39	0.04–3.35	0.39
WBC > 15,000/μL	1.54	0.35–6.69	0.55	16.01	1.76–145.48	0.0137
D-dimer > 0.5 μg/mL	6.26	1.41–27.81	0.0157	1.95	0.39–9.65	0.41
Albumin < 3 g/L	0.72	0.14–3.67	0.69	0.34	0.03–3.48	0.36
		**Organ Failure**			**Death**	
**Covariate**	**OR**	**95% CI**	***p* value**	**OR**	**95% CI**	***p* value**
Age > 65 years	0.99	0.32–3.03	0.99	2.07	0.62–6.91	0.23
Male gender	2.49	0.88–7.04	0.08	1.67	0.61–4.56	0.31
Comorbidities	0.33	0.03–3.16	0.34	2.71	0.31–23.04	0.36
Severe/critical COVID	8.59	2.54–28.98	0.0005	25.19	3.18–199.5	0.002
CCI ≥ 5	0.86	0.19–3.80	0.84	1.63	0.47–5.60	0.43
Hgb < 9 g/dL	0.84	0.14–4.82	0.85	0.17	0.01–2.00	0.15
WBC > 15,000/μL	0.54	0.06–4.65	0.57	4.93	0.71–33.93	0.10
D-dimer > 0.5 μg/mL	5.50	0.50–59.58	0.16	1.82	0.45–7.31	0.39
Albumin < 3 g/L	0.00	0.00–0.00	0.99	6.56	0.87–49.51	0.06

**Table 5:** The outcomes of CDI patients with mild/moderate versus severe/critical COVID-19.

	Univariate Analysis			Multivariate Analysis		
	OR	95% CI	*p* Value	OR	95% CI	*p* Value
Death	24.66	3.12–194.56	0.002	16.40	1.97–136.38	0.009
Organ failure	8.36	2.49–27.98	0.0006	4.97	1.36–18.12	0.01
MOF	4.11	0.45–36.75	0.20	2.88	0.30–27.32	0.35
Prolonged hospitalization	3.41	1.41–8.28	0.006	3.79	1.32–10.84	0.01

OR: odds ratio; CI: confidence interval; MOF: multiple organ failure.

**Table 6:** Multivariate analysis of risk factors for poor prognosis in CDI alone group.

		ICU Admission			Longer Hospital Stays	
Covariate	OR	95% CI	*p* Value	OR	95% CI	*p* Value
Age > 65 years	0.22	0.02–1.62	0.13	2.48	0.76–8.06	0.12
Male gender	2.16	0.44–10.60	0.34	0.59	0.22–1.54	0.28
Comorbidities	0.41	0.02–6.32	0.52	1.93	0.55–6.71	0.29
CCI ≥ 5	5.27	1.31–17.33	0.02	0.30	0.08–1.13	0.05
Hgb < 9 g/dL	0.14	0.01–1.57	0.11	0.17	0.06–0.46	0.0004
WBC > 15,000/μL	5.16	2.40–9.81	0.003	1.79	0.64–5.00	0.26
		**Organ Failure**			**Death**	
**Covariate**	**OR**	**95% CI**	***p*** **value**	**OR**	**95% CI**	***p*** **value**
Age > 65 years	0.08	0.01–0.50	0.006	0.25	0.03–1.66	0.15
Male gender	2.80	0.83–9.46	0.09	2.06	0.45–9.42	0.34
Comorbidities	0.55	0.09–3.05	0.49	0.54	0.04–7.21	0.64
CCI ≥ 5	11.88	1.75–80.66	0.01	10.93	1.16–102.98	0.03
Hgb < 9 g/dL	0.20	0.04–0.93	0.04	0.16	0.01–1.60	0.12
WBC > 15,000/μL	3.97	1.27–12.41	0.01	7.19	1.49–34.67	0.01

ICU: intensive care unit; OR: odds ratio; CI: confidence interval; CCI: Charlson comorbidity index; Hgb: hemoglobin; WBC: white blood cells.

## Data Availability

Not applicable.
